# Endothelial Dysfunction in Acute Myocardial Infarction: A Complex Association With Sleep Health, Traditional Cardiovascular Risk Factors and Prognostic Markers

**DOI:** 10.1002/clc.70080

**Published:** 2025-01-28

**Authors:** Mohamed Ali Hbaieb, Salma Charfeddine, Tarak Driss, Laurent Bosquet, Benoit Dugué, Ahmed Makni, Mouna Turki, Leila Abid, Omar Hammouda

**Affiliations:** ^1^ Laboratory "Mobilité, Vieillissement, Exercise (MOVE) (UR20296)", Faculty of Sport Sciences University of Poitiers Poitiers France; ^2^ Research Laboratory, Molecular Bases of Human Pathology, LR19ES13, Faculty of Medicine University of Sfax Sfax Tunisia; ^3^ High Institute of Sport and Physical Education University of Sfax Sfax Tunisia; ^4^ Cardiology Research Unit, Hédi Chaker University Hospital, Faculty of Medicine University of Sfax Sfax Tunisia; ^5^ Interdisciplinary Laboratory in Neurosciences, Physiology and Psychology: Physical Activity, Health and Learning (LINP2), Faculty of Sport Sciences Paris Nanterre University Nanterre France

**Keywords:** acute myocardial infarction, cardiorespiratory fitness, cardiovascular risk factors, endothelial function, lifestyle behavior, prognosis, sleep health

## Abstract

**Background:**

Endothelial function (EndFx) is a core component of cardiovascular (CV) health and cardioprotection following acute myocardial infarction (AMI) treated with primary percutaneous coronary intervention (PCI).

**Hypothesis:**

AMI patients experience endothelial dysfunction (EndDys), associated with traditional CV risk factors and sleep patterns. EndFx may also predict short and mid‐term outcomes.

**Methods:**

EndFx was assessed in 63 patients (56.2 ± 7.6 years) using the Endothelium Quality Index (EQI). Sleep quality and quantity were evaluated using objective (actigraphy) and subjective (Pittsburgh Sleep Quality Index questionnaire) measures. Cardiorespiratory fitness was quantified through the 6‐min walking test. Cardiac function was assessed using the left ventricular ejection fraction.

**Results:**

Following AMI, patients tended to experience EndDys (EQI = 1.4 ± 0.7). A severe EndDys was observed in 23.8% of patients (*n* = 15), while a mild EndDys was present in 63.49% (*n* = 40). Furthermore, EndDys was significantly associated with traditional CV risk factors (i.e., low physical activity level [12.8%], age [−4.2%], and smoking [−0.7%]) (R^2^ adjusted = 0.50, *p* < 0.001). Patients with EndDys had poor sleep quality (*p* = 0.001) and sleep efficiency (*p* = 0.016) compared to healthy persons. Patients with severe EndDys exhibited lower cardiorespiratory fitness compared to those with healthy EndFx (*p* = 0.017). Furthermore, during a follow‐up period (nearly 4 months) following PCI, major adverse cardiac events were observed in four patients with severe EndDys.

**Conclusions:**

Our results emphasize the importance of adequate sleep and an active lifestyle, notably physical activity practice, as modifiable elements to enhance EndFx, which is regarded as a predictive tool following AMI. However, other factors remain to be elucidated as predictors of CV risk.

**Trial Registration:**

The study protocol was registered in the Pan African Clinical Trial Registry under the trial ID: PACTR202208834230748.

## Summary

- Endothelial function is a crucial aspect of cardiovascular (CV) health, significantly contributing to cardioprotection following an acute myocardial infarction. It is well‐established that traditional CV risk factors, such as low physical activity levels and sedentary behavior, adversely affect CV health. Recently, guidelines have also recognized sleep as an important lifestyle factor impacting CV disease progression.

- Our study aimed to investigate the influence of traditional CV risk factors and sleep health on endothelial function following acute myocardial infarction. Additionally, we hypothesized that endothelial function could predict cardiorespiratory fitness, cardiac function, and the severity of coronary artery disease.

- Our findings revealed that poor sleep health and low physical activity level were the main lifestyle habits associated with endothelial dysfunction. Moreover, endothelial dysfunction was linked to low cardiorespiratory fitness considered a marker of poor prognosis.

- This study emphasizes the importance of promoting healthy lifestyle habits, such as maintaining an active lifestyle and practicing good sleep hygiene, to enhance endothelial function and improve prognosis following an acute myocardial infarction.

## Graphical abstract

Patients following AMI tend to experience endothelial dysfunction that was linked principally to poor sleep quality and physical inactivity lifestyle. Moreover, endothelial function is a prognosis marker of exercise capacity and major adverse acute cardiac events (MAACE). Improving sleep quality and practicing physical activity are essential for enhancing endothelial function considered as a predictive tool to ensure better prognosis and prevent MAACE following AMI.

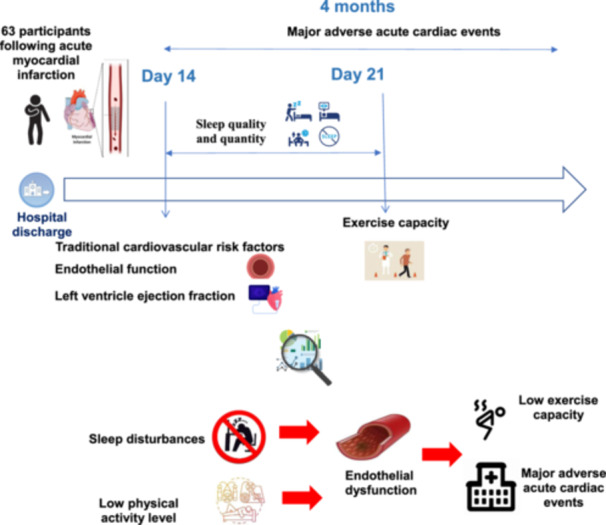

**Abbreviations**
AMI: acute myocardial infarction
CV: cardiovascular
EndDys: endothelial dysfunction
EndFx: endothelial function
EQI: Endothelium Quality Index
PCI: percutaneous coronary intervention
PSQI: Pittsburg Sleep Quality Index
6mwt: 6‐min walking test

## 1 Introduction

The endothelium is a single layer of cells that covers the interior of major and minor vessels. It is an interface between the vessel walls and the circulating blood. Endothelial cells play an active role in regulating vascular tone through responses induced by vasodilator and vasoconstrictive stimuli. Furthermore, the endothelium is involved in angiogenesis, hemostasis control, and maintaining blood fluidity by preventing platelet and leukocyte activation [1].

In recent decades, endothelial function has been widely used as a research tool and has gained prominence, particularly in cardiovascular disease (CVD) pathogenesis. It has been recognized as a valuable marker for prognosis following acute myocardial infarction (AMI) due to its role in regulating vascular health [2].

Furthermore, endothelial function is a key component of cardioprotection, which gathers all measures and interventions to prevent, attenuate, and repair myocardial injury [3]. Therefore, cardioprotective strategies aim to reduce infarct size and recover ventricular function after reperfusion of the ischemic area following AMI.

In this context, a healthy endothelium, particularly through endothelial‐derived nitric oxide (NO) production, provides a cardioprotective effect that limits ischemia‐reperfusion (IR) injury. Moreover, elevated NO production has been observed to suppress endothelial cellsinflammation and to limit infarct size [4]. Conversely, endothelial dysfunction, marked by reduced NO production and increased sensitivity to vasoconstrictor substances such as endothelin‐1 (ET‐1) and pro‐inflammatory compounds, heightens the risk of IR injury [5]. Indeed, endothelial dysfunction‐induced atherosclerosis contributes to the impairment of barrier function associated with inflammatory response. This consequently promotes the heightened infiltration of low‐density lipoprotein and leukocyte extravasation into the vessel walls, ultimately leading to plaque instability [6].

Endothelial function is a strong predictor of CVD and is a major component of CV health. It is well‐established that CV health encompasses modifiable lifestyle behaviors, including physical activity levels, diet, and smoking [7]. Evidence demonstrates that low physical activity levels and sedentary behavior are associated with metabolic disorders and poor CV health, including endothelial dysfunction, which increases the risk of CVD [8]. To stem poor lifestyle habits, a new field of medicine was established “Lifestyle Medicine” which aims to prevent and treat chronic disease using behavioral techniques and therapies. Cardiologists are drawn to lifestyle medicine practices because of their involvement in three subspecialty areas: behavioral cardiology, preventive cardiology, and cardiac rehabilitation [9]. In this line, an initiative was induced by the American Heart Association named “Life's Simple Seven” to address unhealthy lifestyle behaviors and to advance preventive cardiology [10]. Despite the efforts made to reduce the incidence of CVD, behavioral counseling targeting tobacco use, healthy diet, and physical activity practice was reduced in low and middle‐income countries [11].

Recently, the American Heart Association added sleep to its list of traditional risk factors in a 2022 update known as “Life's Essential Eight,” ranking it as the eighth major component of CV health [12]. Interestingly, sleep is considered a novel independent risk factor for atherosclerotic CVD [13]. Sleep is a complex and multidimensional parameter, encompassing various metrics such as sleep duration and sleep quality. It plays a key role in maintaining an optimal body system by promoting CV health, regulating hormones, strengthening the immune system, and consolidating cognitive functions [14].

Otherwise, most physiological processes, including heart rate, heart rate variability, blood pressure, platelet activity, vagal modulation, cardiomyocyte function, and endothelial cells display a circadian rhythm regulated mainly by sleep/wake and fasting/feeding cycles [15]. A clear link between human synchronization of the circadian clock and CV health has been established [16]. In this line, the daily light/dark pattern was considered a new modifiable lifestyle and a synchronizer of the circadian clock [17].

It has been demonstrated that coronary artery disease (CAD) patients usually suffer from sleep disturbances with reduced levels of melatonin and desynchronization of the circadian clock [18]. Yet, sleep disorders are associated with increased levels of inflammatory markers, endothelial dysfunction, and reduced cardiac vagal modulation leading to CAD [19].

The connections between endothelial function and sleep patterns have received little attention in cardiology studies. Therefore, the main objective of this study was to investigate whether poor sleep quality and quantity were associated with endothelial dysfunction in CAD patients after AMI. In addition, a secondary purpose of this study was to investigate whether the traditional CV risk factors were associated with endothelial dysfunction. This study was also implemented to test the hypothesis that endothelial function could be a prognostic indicator for short (cardiorespiratory fitness, left ventricular ejection fraction (LVEF) and severity of the CAD status) and mid (major adverse acute and chronic events (MAACE)) terms.

## 2 Methods

### 2.1 Study Design

Before enrollment in the study, all patients were thoroughly informed about the study's protocol and provided written informed consent. Ethical approval for this study was obtained from the local Ethic and Investigation Committee (CPP SUD N° 0331/2021). The study protocol was registered in the Pan African Clinical Trial Registry under the trial ID: PACTR202208834230748. This study was conducted according to the Declaration of Helsinki.

### 2.2 Participants

This study enrolled patients with acute ST‐elevation myocardial infarction (STEMI) who underwent a primary percutaneous coronary intervention (PCI) within 120 min of the onset of chest pain. For patients who did not have immediate access to primary PCI, the procedure was performed within 24 h after successful immediate fibrinolysis, following the guidelines of the European Society of Cardiology [20]. Patients with non‐STEMI included in this study underwent a primary PCI within 24 h.

### 2.3 Echocardiography Parameters

LVEF was measured by 2D echocardiography (EPIQ CVx model; Philips, Bothell, WA, USA) on admission and 24 h post‐primary PCI.

### 2.4 Endothelial Function

Endothelial function was assessed between the second and the third week post‐primary PCI using an E4‐diagnose device (Polymath Company, Tunisia) that fully automates post‐occlusion hyperemia protocol. The E4‐diagnose is a noninvasive, high‐resolution (0.002°C) skin temperature measuring device composed of two finger temperature sensors, an integrated wrist cuff placed on the dominant forearm, and a portable micro unit controller. Notably, measurements in the nondominant hand serve as an internal control. A dedicated PC software was used to view, store, and export data. All measurements were performed in the morning, in a dimmed and quiet room with an ambient temperature between 22°C and 24°C. Patients were fasting for at least 8 h with no smoking and no heavy physical activity at least for 4 h and kept in a relaxing sitting position at least 20 min before the test. Before starting the test, finger's temperature must be above 27°C. Baseline temperature was recorded during the first 5‐min period followed by a 5‐min cuff occlusion period. During the occlusion period, the temperature of the dominant hand index finger decreased due to the absence of warm circulated blood. Following the 5‐min cuff occlusion period, the cuff was released and the hyperemic blood flow was restored to the dominant hand causing the “temperature rebound” recorded in the index finger reflecting endothelial function by the Endothelium Quality Index (EQI) [21].

Based on the EQI, endothelial function was classified as follows: EQI > 2: healthy endothelial function, 1 ≤ EQI ≤ 2: mild endothelial dysfunction, EQI < 1: severe endothelial dysfunction [22].

### 2.5 Sleep Quality and Quantity

A wrist‐worn accelerometer, based on motion sensors across three axes, was used to assess objectively sleep quality and quantity (ActiGraph GT3X, ActiGraph Inc, Pensacola, FL, USA). All patients wore the accelerometer during the third week post‐primary PCI. All accelerometers were initialized to a sampling rate of 30 Hz. The start time was set to midnight on the first day and the stop time was set to midnight on the seventh day. Actilife software (version 6.8.1; ActiGraph LLC, Pensacola, FL, USA) was used for the exportation of data. They were extracted in 60 s epochs to optimize the signal‐to‐noise ratio. It is worth noting that ActiGraph Gt3X has been validated for sleep period detection, as well as periods of wakefulness based on wrist movements during the night. The Cole‐Kripke algorithm was used to measure sleep efficiency, sleep duration, wake‐after‐sleep onset, and total time in bed [23].

The Pittsburg Sleep Questionnaire Index (PSQI) questionnaire was used to complete the objective assessment with a subjective assessment. The PSQI index is the sum of seven components, each equally scored on a 0–3 scale: subjective sleep quality, sleep duration, sleep latency, sleep efficiency, use of sleep medication, sleep disturbances, and daytime dysfunction. Patients with a PSQI index above 5 were considered to have poor sleep quality [24].

### 2.6 Physical Activity Level

The physical activity level was assessed using the Ricci–Gagnon scale (RG; Montreal). It consists of nine items that assess sedentary behavior, leisure physical activity, and daily physical activity with a 5‐point Likert scale. Participants are considered inactive when the sum of the nine items is < 18, and active when this sum is ≥ 18 [25].

### 2.7 Cardiorespiratory Fitness

Distance covered during the 6‐min walking test (6mwt) was considered a proxy for cardiorespiratory fitness. The test was conducted in a 30‐meter hospital corridor during the third week post‐primary PCI. Patients were asked to walk as far as possible for 6 min and to stop in case of palpitations, dizziness, or rapid change in vital signs. Total distance after the 6 min was recorded, as well as perceived exertion with the Borg's scale. Heart rate, blood pressure, and oxygen saturation were measured before and after the test.

### 2.8 Statistical Analyses

The normality of the data distribution was assessed by the Shapiro‐Wilk test. The null hypothesis of an absence of differences between groups was tested through a one‐way ANOVA when data were normally distributed, or with a Kruskal Wallis test when they did not fulfill this condition. A Bonferroni post‐hoc test was used for pairwise comparisons when the null hypothesis was rejected.

The null hypothesis of an absence of difference between groups in categorical variables regarding clinical characteristics was analyzed through the Chi 2 test.

The null hypothesis of an absence of association between relevant parameters was tested through the Pearson test product‐moment correlation when data were normally distributed, or with the Spearman rank order correlation when they were not.

Multivariable regression was used to explore whether certain clinical factors (age, body mass index (BMI), dyslipidemia, diabetes mellitus, HbA1c, smoking, and physical activity level) have an impact on endothelial function, and to identify short‐term outcomes associated with endothelial dysfunction. An α risk of 5% was retained for all analyses. All statistical analyses were performed with SPSS (Statistical Package for the Social Sciences) version 26 (SPSS Inc., Chicago, IL, USA).

## 3 Results

A total of 63 patients with a mean age of 56.2 ± 7.6 years were included in this study following AMI. Their clinical characteristics were summarized in Table 1.

**TABLE 1:** Characteristics of the study population.

Characteristics		Mean ± SD or n (%)
**Cardiovascular risk factors**		
New York Heart Association Class 1		34 (54%)
New York Heart Association Class 2		26 (41.3%)
New York Heart Association Class 3		3 (4.8%)
Body Mass Index (kg/m^2^)		27.06 ± 3.60
Smoking (pack years)		31.52 ± 29.84
High blood pressure		36 (57.1%)
Dyslipidemia		46 (73%)
Triglyceride (mmol/L)		1.28 ± 0.61
Total cholesterol (mmol/L)		3.30 ± 0.94
High‐density lipoprotein (mmol/L)		0.91 ± 0.19
C‐Reactive protein (mg/L)		7.46 ± 8.66
Exercise‐induced asthma		30 (47.6%)
Diabetes mellitus		40 (63.5%)
Glycated hemoglobin (%)		7.03 ± 1.88
Family history of coronary artery disease		32 (50.8)
Physical activity level (Ricci ‐Gagnon Score)		12.36 ± 2.84
**Cardiac function**		
Left ventricular ejection fraction (%)		53.62 ± 9.09
**Severity of obstructive coronary artery disease**		
Single‐vessel coronary artery disease		38 (60.3%)
Double‐vessel coronary artery disease		18 (28.6%)
Triple‐vessel coronary artery disease		7 (11.1%)
**Revascularization**		
Complete revascularization		45 (71.4%)
Incomplete revascularization		18 (28.6%)
**Medication**		
**Platelet aggregation inhibitor**	Aspirin	59 (93.7%)
	Clopidogrel	60 (95.2%)
	Ticagrelor	3 (4.8%)
**Anticoagulant therapy**	Vitamin K antagonist	2 (3.2%)
	Rivaroxaban	0
	Apixaban	0
Statins		57 (90.5%)
Angiotensin‐converting enzyme inhibitors		39 (61.9%)
Angiotensin 2 inhibitors		10 (15.9%)
B‐Adrenergic receptor antagonist		56 (88.9%)
Oral hypoglycemic medications		23 (36.5%)
Insulin therapy		3 (4.8%)
SGLT2 inhibitors		4 (6.3%)
Aldosterone inhibitors		5 (7.9%)
Calcium‐channel antagonist		19 (30.2%)
Nitrates		12 (19%)
Thiazide diuretics		1 (1.6%)
Vessel		1 (1.6%)

*Note:* Data are presented as mean ± Standard deviation (SD) or as number (n) and frequency.

### 3.1 Endothelial Function

A severe endothelial dysfunction was observed in 23.8% of patients (*n* = 15), while a mild endothelial dysfunction was present in 63.49% (*n* = 40). Endothelial function was considered normal in 12.7% of patients (*n* = 8) (Table 2). The mean EQI was 1.4 ± 0.7.

**TABLE 2:** Demographics, clinical characteristics, and medication in endothelial function groups.

Characteristics Endothelial function class	Severe endothelial dysfunction (*n* = 15)	Mild endothelial dysfunction (*n* = 40)	Healthy endothelial function (*n* = 8)
Age (year)	59.10 ± 9.59	55.90 ± 7.35	52.60 ± 6.8
Body mass index (kg/m^2^)	26.80 ± 3.59	27.60 ± 3.62	25.10 ± 3.18
Smoking (pack years)	45.90 ± 42.85	27.20 ± 22.23	26.30 ± 27.04
High blood pressure	10 (66.7%)	21 (52.5%)	5 (62.5%)
C‐Reactive protein (mg/L)	6.2 ± 6.07	7.89 ± 9.16	7.71 ± 10.93
Dyslipidemia	11 (73.3%)	32 (80%)	3 (37.5%)^a^
Triglyceride (mmol/L)	1.44 ± 0.66	1.24 ± 0.61	1.20 ± 0.48
Total cholesterol (mmol/L)	3.28 ± 0.88	3.30 ± 0.1	3.40 ± 0.83
High‐density lipoprotein (mmol/L)	0.96 ± 0.22	0.89 ± 0.19	0.94 ± 0.12
Asthma	9 (60%)	19 (63.3%)	2 (25%)
Diabetes mellitus	11 (73.3%)	24 (60%)	5 (62.5%)
Glycated hemoglobin (%)	7.62 ± 2.17	7.07 ± 1.86	5.75 ± 0.50
Family history of coronary artery disease	7 (46.7%)	20 (50%)	5 (62.5%)
Physical activity level	10.2 ± 1.21	12.4 ± 2.66	16.1 ± 1.73^a^, ^b^
New York Heart Association Class 1	7 (46.7%)	23 (57.5%)	4 (50%)
New York Heart Association Class 2	6 (40%)	16 (40%)	4 (50%)
New York Heart Association Class 3	2(13.3%)	1 (2.5%)	0 (0%)
6‐min walking test (meter)	368.90 ± 98.77	438 ± 73.49	448.50 ± 71.33^b^
Left ventricular ejection fraction (%)	53.20 ± 8.24	53.50 ± 9.21	55 ± 11.02
Single‐vessel coronary artery disease	10 (66.7%)	23 (57.5%)	5 (62.5%)
Double‐vessel coronary artery disease	3 (20%)	13 (32.5%)	2 (25%)
Triple‐vessel coronary artery disease	2 (13.3%)	4 (10%)	1 (12.5%)
Complete revascularization	12 (80%)	27 (67.5%)	6 (75%)
Incomplete revascularization	3 (20%)	13 (32.5%)	2 (25%)
Aspirin	13 (86,7%)	38 (95%)	8 (100%)
Clopidogrel	14 (93.3%)	38 (95%)	8 (100%)
Ticagrelor	1 (6.7%)	2 (5%)	0
Vitamin K antagonist	1 (6.7%)	0	1 (12.5%)
Rivaroxaban	0	0	0
Apixaban	0	0	0
Statins	12 (80%)	37 (92.5%)	8 (100%)
Angiotensin‐converting enzyme inhibitors	6 (40%)	26 (66.7%)	7 (87.5%)
Angiotensin 2 inhibitors	3 (20%)	6 (15%)	1 (12.5) %
B‐Adrenergic receptor antagonist	13 (86.7%)	36 (90%)	7 (87.5%)
Oral hypoglycemic medications	8 (53.3%)	12 (30%)	3 (37.5%)
Insulin therapy	2 (13.3%)	1 (2.5%)	0
SGLT2 inhibitors	0	4 (10%)	0
Aldosterone inhibitor	0	5 (12.5%)	0
Calcium‐channel antagonist	4 (26.7%)	14 (35%)	1 (12.5%)
Nitrates	4 (26.7%)	6 (15%)	2 (25%)
Thiazide diuretics	1 (6.7%)	0	0
Vessel	1 (6.7%)	0	0

*Note:* Data are presented as mean ± Standard deviation (SD) or as number (n) and frequency.

^a^ Different from patients with mild endothelial dysfunction.

^b^ Different from patients with severe endothelial dysfunction.

### 3.2 Sleep Patterns

Sleep parameters in patients following AMI were summarized in Table 3.

**TABLE 3:** Sleep quality and quantity in endothelial function groups.

Characteristics Endothelial function class	Overall	Severe endothelial dysfunction (*n* = 15)	Mild endothelial dysfunction (*n* = 40)	Healthy endothelial function (*n* = 8)
Sleep duration (min)^c^	412.5 ± 82.60	397.7 ± 85.48	414.3 ± 83.88	431.6 ± 75.93
Sleep efficiency (%)^c^	87.01 ± 8.24	86.7 ± 4.60	86.2 ± 9.64	91.9 ± 3.01^a^, ^b^
Total time in bed (min)^c^	479.4 ± 87.9	461.7 ± 105.9	484.9 ± 80.9	485.2 ± 93.4
Wake after sleep onset (min)^c^	67.9 ± 48.73	62.4 ± 30.7	67.97 ± 48.7	77.79 ± 75.9
Pittsburgh Sleep Quality Index	10.3 ± 3.64	12.9 ± 3.26	9.93 ± 3.32	7.38 ± 3.07^a^, ^b^

*Note:* Data are presented as mean ± Standard deviation (SD) or as number (n) and frequency.

^a^ Different from patients with mild endothelial dysfunction.

^b^ Different from patients with severe endothelial dysfunction.

^c^ Assessed using accelerometry.

### 3.3 Endothelial Function and Sleep Patterns

**FIGURE 1:**
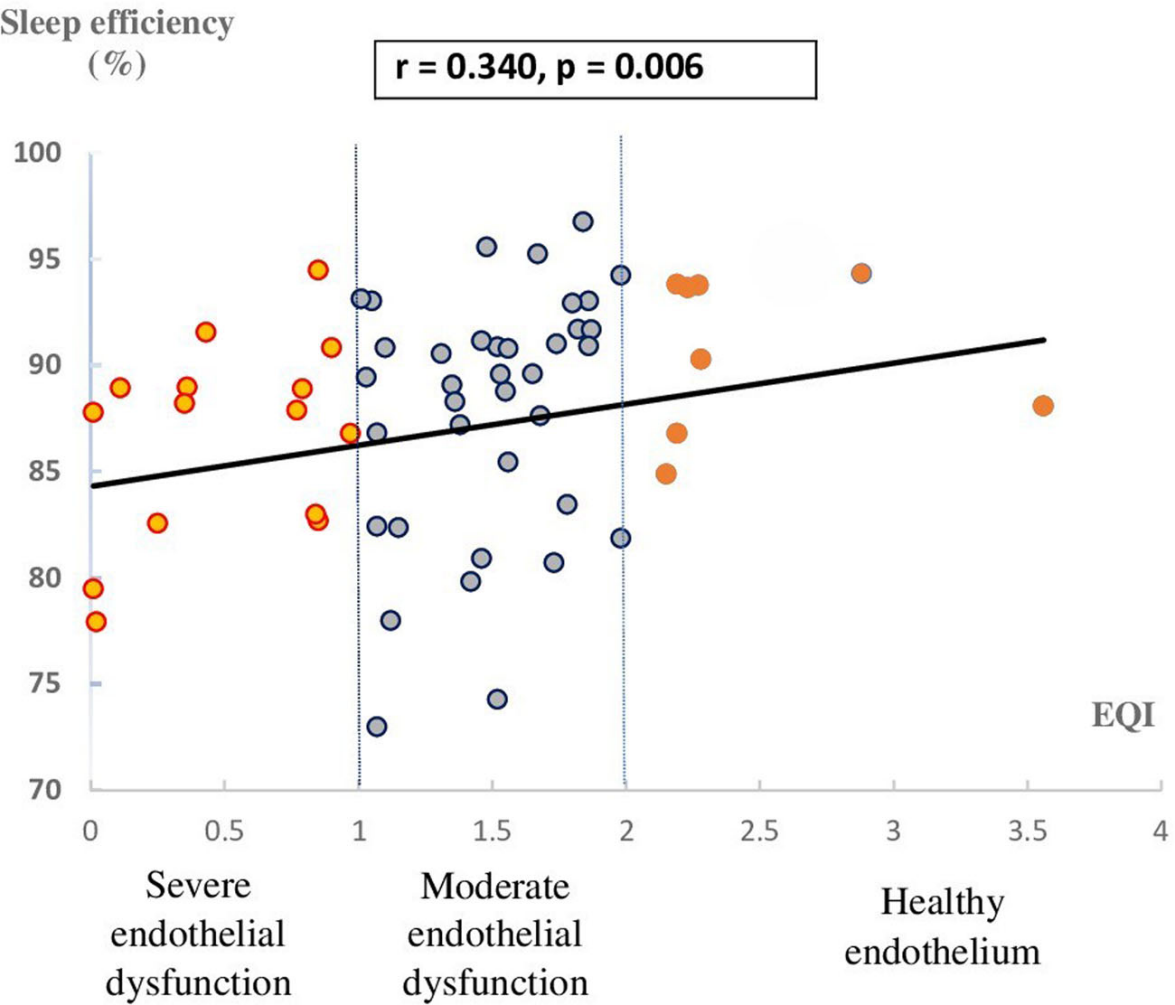
Association between sleep efficiency and Endothelium Quality Index. EQI, Endothelium Quality Index.

**FIGURE 2:**
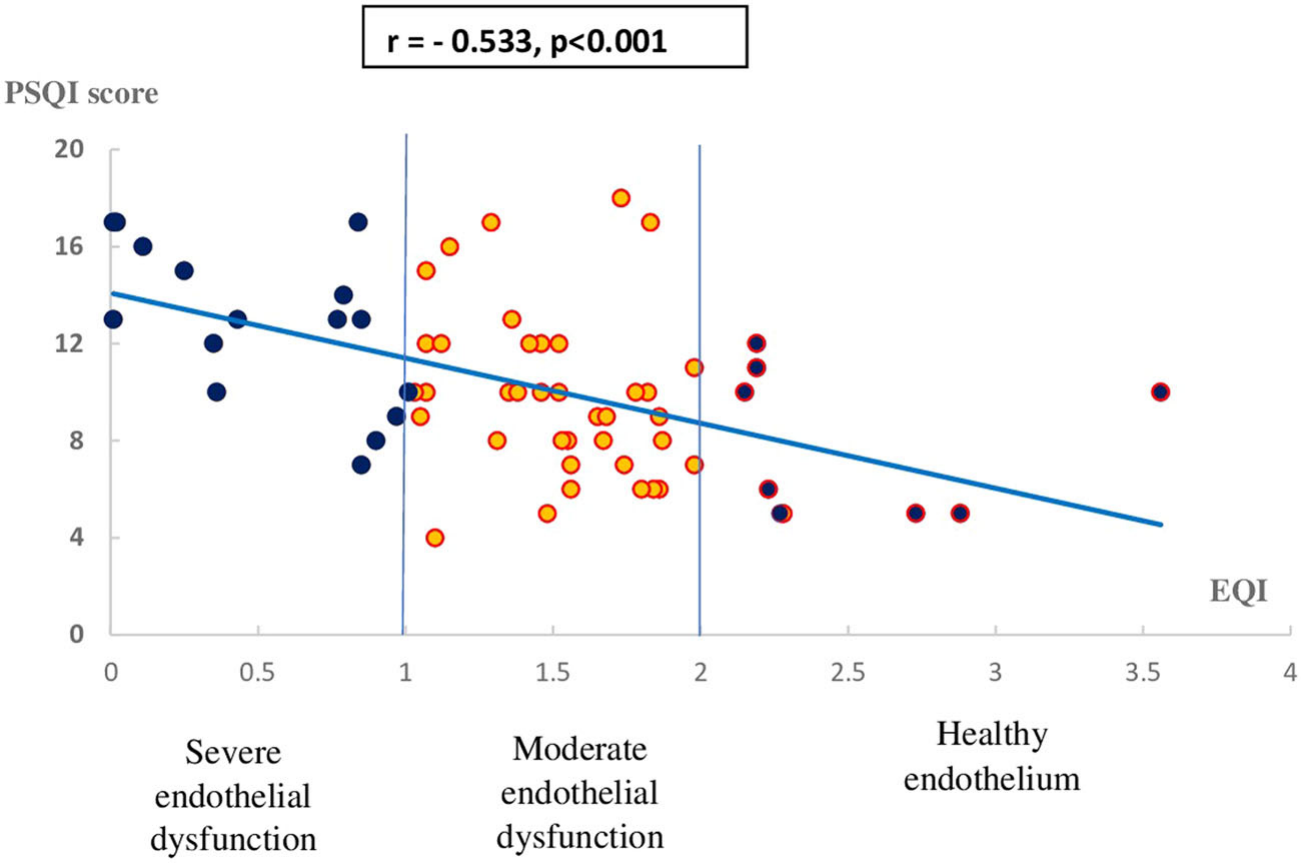
Inverse correlation between PSQI score and endothelium quality index. PSQI, Pittsburgh sleep quality index. EQI, endothelium quality index.

An association was found between EQI and sleep efficiency (r = 0.34, *p* = 0.006) (Figure 1), as well as between EQI and PSQI scores (r = ‐ 0.53 *p* < 0.001) (Figure 2).

Kruskal Wallis test revealed a difference in sleep efficiency (H = 6.013, *p* = 0.049, η2 = 0.07) and subjective sleep quality (H = 13.231, *p* = 0.001, η2 = 0.19) between endothelial function categories (Table 3). Bonferroni post‐hoc analysis revealed that patients with healthy endothelial function had better subjective sleep quality (*p* = 0.001) and better sleep efficiency than patients with severe endothelial dysfunction (*p* = 0.016).

No difference was found between endothelial function groups in sleep duration, total time in bed, and wake after sleep onset (Table 3).

### 3.4 Effect of Traditional CV Risk Factors on Endothelial Function

Multiple regression analyses showed that approximately 56.4% of the variance in endothelial function is related to CV risk factors (F _(6,56)_ = 8.749, R^2^ adjusted = 0.50, *p* < 0.001). Based on the analysis, the primary risk factors believed to influence endothelial function are identified as physical activity level, age, and smoking as presented in Table S1.

We found a difference in physical activity levels between groups (H = 23.22, *p* < 0.001, η2 = 0.35). Patients with healthy endothelial function were more active than patients presenting endothelial dysfunction, whatever its severity (*p* < 0.01).

EQI was inversely correlated with concentration of triglycerides (r = −0.275, *p* = 0.029) and HbA1c (r = −0.315, *p* = 0.012) in blood.

### 3.5 Short‐Term Outcomes

Multiple regression analyses showed that endothelial dysfunction affected cardiorespiratory fitness following AMI (F_(3,59)_ = 3.185, R^2^ adjusted =0.096, *p* = 0.03). However, the severity of CAD and LVEF were not affected by endothelial dysfunction as shown in Table S2.

One‐way ANOVA test revealed a significant difference in 6mwt between the endothelial function groups (F_(2, 60)_ = 4.56, *p* < 0.014, η2 = 0.13) (Table 2). Bonferroni post‐ hoc test revealed that patients with healthy endothelial function exhibited higher cardiorespiratory fitness in comparison to those with severe endothelial function (*p* = 0.017). Moreover, a significant correlation was found between EQI and 6mwt (r = 0.291, *p* = 0.021) using the Spearman test (Figure S1).

### 3.6 Mid‐term Follow‐Up

The average duration of mid‐term follow‐up for MAACE was 4 months. Out of the 63 patients, four individuals encountered rehospitalization due to acute coronary syndrome. All these patients exhibited severe endothelial dysfunction, as indicated by EQI values of 0.78, 0.01, 0.97, and 0.99, respectively. Among these cases, two patients exhibited double‐vessel disease, while two had triple‐vessel disease. Moreover, one patient with an EQI value of 0.28 and incompletely revascularized triple‐vessel CAD died 4 weeks after AMI.

## 4 Discussion

In this study, endothelial function was evaluated in the context of AMI treated with primary PCI. It is a practice not commonly implemented in patient management. It is well established that endothelial dysfunction plays a crucial role in atherogenesis and the development of CVD. The findings of this pilot study showed that 87.3% of the patients following AMI tend to experience endothelial dysfunction. Our results are consistent with a previous study reporting that endothelial dysfunction, assessed using the reactive hyperemia peripheral artery tonometry method, was present in 50% of the patients with AMI after primary PCI [26]. Endothelial dysfunction can cause arterial vasospasm that may reduce blood flow and aggravate arteriosclerotic plaque formation leading to acute coronary syndrome [27]. Indeed, myocardial ischemia arises when the metabolic requirements of the myocardium are not sufficiently fulfilled due to decreased blood flow [28]. However, it is not well understood whether endothelial dysfunction is already impaired before AMI or if it was induced by IR. It has been well demonstrated that a sudden supply of oxygen during reperfusion induces a more proinflammatory response in endothelial cells already caused by ischemia. The overproduction of reactive nitrogen species, exacerbation of oxidative stress associated with the overproduction of reactive oxygen species that reduce NO bioavailability, and transmigration of leukocytes through the endothelium are the major mediators contributing to endothelial dysfunction [28].

To the best of our knowledge, limited studies have explored the relationship between sleep components and CV health in cardiology. This study seeks to address this gap in the literature by examining sleep quality and quantity in patients following AMI and investigating its potential link with CV health indicators such as endothelial function. Sleep health has been recognized as a modifiable CV risk factor and a novel component of CV health associated with a broad spectrum of CV events, including AMI [12]. In this context, a most recent review and a large meta‐regression analysis of 40 prospective cohort studies showed that poor sleep quality and insufficient or excessive sleep duration were associated with increased systemic inflammation and reduced cardiac vagal modulation, which could be linked to a higher risk of CVD and all‐cause mortality [29, 30].

The present study showed that patients with endothelial dysfunction tend to experience poor sleep quality as compared to those with healthy endothelial function. These results align with a previous study involving 684 healthy subjects revealing an inverse association between endothelial function assessed through flow mediation dilation (FMD) and PSQI score, along with the Epworth Sleepiness Scale score [31]. Furthermore, a more recent meta‐analysis emphasized the link between endothelial function and sleep disturbances, highlighting that lower FMD was linked to shorter sleep duration and circadian misalignment often observed in shift workers [32]. Notably, none of the studies establishing the association between sleep patterns and endothelial function included patients with CVD. Therefore, including patients with CVD underscores the originality of our current study.

Sleep health has been recognized as a core component of CV health. Notably, during normal sleep, heart rate and blood pressure are decreased. Furthermore, the dominance of either the sympathetic or parasympathetic nervous system characterizes each sleep phase [17]. Consequently, poor sleep health elicited an imbalance in the autonomic nervous system and neurohormones with overactivity of sympathetic tone that plays a crucial role in the development of hypertension by increasing vascular resistance [14]. Notably, high blood pressure exerts excessive mechanical stress on endothelial cells and exacerbates oxidative stress that decrease the bioavailability of NO contributing to endothelial dysfunction.

In this line, the link between sleep disturbances and endothelial dysfunction could be explained by the presence of peripheral clocks within endothelial and vascular smooth muscle cells, which are controlled by the central biological clock located in the suprachiasmatic nuclei. These clocks regulate the circadian oscillation of NO and ET‐1 [33]. The synchronization between the central clock and peripheral clocks located in cardiac tissue is controlled by melatonin, a key circadian hormone produced mainly by the pineal gland. Melatonin has a potential role in maintaining and stabilizing circadian rhythms [34]. Notably, circadian misalignment associated with sleep disturbances is caused by reduced levels of melatonin observed in CVD patients [18]. Consequently, the balance between NO and ET‐1 is disrupted, thereby impairing endothelial function. In addition, melatonin has a cardioprotective effect against IR injury, which is characterized by the proinflammatory response and exacerbation of oxidative stress contributing to atherosclerosis and endothelial dysfunction [35]. Numerous studies have confirmed that melatonin is a potent free radical scavenger and has a powerful capacity to activate antioxidant enzymes [36].

Several studies have shown that sleep disorders can affect lifestyle behaviors, including diet timing and physical activity practice, contributing to an increased risk of CVD [37].

In the literature, a limited number of studies have established a clear link between lifestyle behaviors such as physical activity level, considered a modifiable CV risk factor, and endothelial function in patients with CVD. In the current study, multiple regression analysis revealed a significant association between low physical activity levels and endothelial dysfunction. In addition, comparison group analysis showed that active patients had better endothelial function than sedentary patients. Accordingly, a study among 2363 patients with prediabetes and type 2 diabetes demonstrated that sedentary behavior was linked to increased low‐grade inflammation biomarkers (i.e., C‐reactive protein, soluble intercellular adhesion molecule‐1, IL‐6, and TNF‐α), as well as endothelial dysfunction biomarkers [38]. In this line, a more recent study among chronic kidney disease patients showed that endothelial dysfunction biomarkers increased with sedentary behaviors associated with increased oxidative stress leading to reduced NO bioavailability [39, 40]. Conversely, higher levels of physical activity and the regularity of moderate to vigorous intensity activity displayed an inverse correlation with endothelial dysfunction and low‐grade inflammation biomarkers [41]. These findings underscore the cardioprotective effects of physical activity, which enhances endothelial function by inducing shear stress that increases the bioavailability of NO [42]. Supporting this, a meta‐analysis revealed that exercise training improved endothelial function, particularly in patients with CVD [43]. Additionally, it was well demonstrated that individuals who practice physical activity even under the recommended threshold (150 min of moderate aerobic physical activity per week, 75 min of vigorous aerobic physical activity per week, or a combination of both) had a lower mortality risk compared to sedentary individuals [44]. Additionally, a most recent study demonstrated that engaging in moderate to vigorous physical activity above the recommended threshold can mitigate the adverse effects of sleep disorders, as well as all‐cause and CV mortality [45]. Interestingly, increasing daily step counts was associated with improved CV health outcomes [46]. In sum, patients following AMI should reduce the sitting time associated with detrimental effects and practice moderate to vigorous physical activity coupled with resistance training contributing to greater clinical effects, particularly on CV health [47].

In this study, an inverse correlation was observed between EQI and HbA1c, as well as triglyceride levels. In this line, a study among patients with type 2 diabetes mellitus demonstrated the presence of a U‐shaped pattern of association between HbA1c and endothelial function. Endothelial dysfunction was observed in patients with HbA1c below 6.5% and above 7% [48]. Furthermore, our results align with a most recent review highlighting an association between dyslipidemia and endothelial dysfunction [49]. Additionally, our findings indicated that age and smoking were significant factors linked to endothelial dysfunction. This is consistent with evidence showing that older individuals and smokers are more prone to endothelial dysfunction [50, 51]. These traditional CV risk factors are linked to an exacerbation of oxidative stress, which is the primary cause of reduced bioavailability of NO. The rapid scavenging of NO by reactive oxygen species can lead to the formation of highly prooxidant compounds, which could be responsible for endothelial dysfunction [52].

It has been established that endothelial function not only reflects poor prognosis but also predicts outcomes after acute coronary syndrome. In the present prospective study, endothelial dysfunction was associated with the occurrence of MAACE such as recurrent acute coronary syndrome and CV death during the mid‐term follow‐up. These findings confirm our hypothesis that endothelial function is considered a prognosis marker in CAD patients. In this regard, a strong connection between assessed endothelial dysfunction and an elevated risk of in‐stent restenosis following primary PCI has been observed [53]. Furthermore, among patients with non‐STEMI, endothelial dysfunction is linked to an increased risk of CV events within 14 months, particularly in the presence of diabetes mellitus [54].

While numerous studies have focused on the association between endothelial dysfunction and MAACE in the mid‐term period after cardiac events, there is a growing interest in exploring its impact on short‐term clinical outcomes following AMI such as cardiorespiratory fitness, CAD status, and LVEF. These outcomes are considered a crucial prognostic marker following AMI.

The findings of our study showed that endothelial function was strongly associated with exercise capacity. Cardiorespiratory fitness reflects the synergetic functioning of cardiac function, pulmonary ventilation, and the capacity of vascular function to deliver and unload oxygen. It has been demonstrated that low exercise capacity, which is associated with CV risk factors such as obesity and low physical activity levels, is linked to bad prognosis and increased mortality risk [55]. In this line, reduced exercise tolerance evaluated with 6mwt is considered a short‐term clinical outcome related to increased risk of death and reinfarction in patients with AMI [56]. Reduced exercise capacity after primary PCI is a result of the overproduction of reactive oxygen species, which in turn leads to muscle cell damage. Our results are comparable with previous findings among patients with chronic obstructive pulmonary disease indicating a significant correlation between endothelial function evaluated with FMD and 6mwt [57]. During exercise, NO bioavailability plays a crucial role in increasing blood flow to exercising skeletal muscle, reducing blood pressure, and more importantly, reducing oxygen cost at a given workload [58]. Moreover, while NO plays a crucial role in regulating glucose uptake during exercise, endothelial dysfunction is linked with energy metabolism disorders that impair exercise tolerance [59].

Alongside cardiorespiratory fitness, the severity of CAD was assessed due to its relative importance in prognosis following AMI. In this population, 60.3% of patients had single‐vessel disease and 39.6% presented multi‐vessel disease. Similarly, a previous study has found that 40.1% of AMI patients presented multi‐vessel disease [60]. However, our study did not find a significant link between the severity of CAD and endothelial function. The absence of a significant correlation may be attributed to the relatively infrequent presence of multi‐vessel disease, especially cases of triple‐vessel disease, within the study's participants.

Regarding cardiac function, we noted the absence of a link between endothelial function and LVEF following AMI. Ischemic heart failure characterized by reduced LVEF, which implies cardiac dysfunction, is a short‐term clinical outcome that occurs in approximately 30%–40% of patients following AMI. Moreover, it carries a poor cardiac prognosis even after primary PCI [61]. Additionally, endothelial dysfunction is widely linked to an increased risk of mortality in patients with heart failure [62]. Recently, it was demonstrated that patients with ischemic heart failure exhibited endothelial dysfunction with lower FMD [63]. Endothelial dysfunction is widely associated with fibrosis considered a key element in myocardial remodeling leading to a reduction in vascular compliance, imposing elevated loads on the left ventricle contributing to left ventricle stiffness and heart failure [64]. In this line, a linear improvement in FMD was related to an improvement in LVEF [63]. The reduced number of patients with reduced LVEF can explain our results.

The findings of this study have significant clinical implications for enhancing patient management following AMI. A crucial step is evaluating various sleep metrics, including quality and quantity, alongside traditional CV risk factors. Additionally, assessing endothelial function, an often‐overlooked clinical measure following AMI, is recommended. To improve prognosis and prevent MAACE, it is essential to promote better sleep health. This can be achieved through key lifestyle practices [17] (i.e., sleeping between 7 and 9 h per night, maintaining a regular bedtime and sleep duration, increasing daylight exposure, and reducing evening light exposure) that regulate light and dark patterns, thereby reducing circadian desynchronization and enhancing endothelial function. Furthermore, post‐AMI patients are encouraged to adhere to guideline‐recommended supervised physical activity levels to optimize CV health. In addition, it is crucial to better control dyslipidemia and glycemia, as well as smoking cessation to mitigate endothelial function and MAACE. This study addresses detrimental lifestyle habits, by incorporating behavioral therapies and promoting a healthy and active lifestyle. This approach is particularly relevant for implementing “Lifestyle Medicine” in low and middle‐income countries, serving as a cost‐effective strategy for primary and secondary prevention.

### 4.1 Study Limitations

Our study has several limitations. It was a single‐center study presenting a relatively small number of patients which limits the generalizability of the findings, necessitating future research with larger cohorts to validate these results. Additionally, the study's duration of mid‐term follow‐up for MAACE was relatively short at approximately 4 months, which may not sufficiently capture mid‐term outcomes. Furthermore, we evaluated endothelial function using the E4‐Diagnose device, which is not the gold standard for assessing endothelial function. Further studies should use FMD to confirm the link between endothelial function and measured outcomes. Additionally, the majority of the patients had preserved LVEF, which may not adequately represent the variability in endothelial dysfunction across different LVEF levels. Future studies should include a more diverse patient population, especially those with heart failure, to better understand the relationship between LVEF and endothelial dysfunction. Additionally, further investigation should measure oxidative stress and additional measurements for inflammatory biomarkers such as IL6, which is considered a prominent mediator of sleep disturbances, physical inactivity, and endothelial dysfunction. In our study, we were unable to measure oxidative stress biomarkers and IL‐6 due to the high cost and lack of funding.

## 5 Conclusion

Patients who have experienced AMI and received primary PCI displayed endothelial dysfunction. This impairment was linked not only to traditional CV risk factors, such as low levels of physical activity but also to poor sleep quality. Moreover, endothelial dysfunction affected exercise capacity and it was associated with MAACE. These findings emphasize the maintenance of an active lifestyle and addressing sleep‐related issues to potentially improve endothelial function considered as a strong prognosis marker that predicts short and mid‐term outcomes.

## Ethics Statement

Ethical approval for this study was obtained from the local Ethic and Investigation Committee (CPP SUD N° 0331/2021). This study was conducted according to the Declaration of Helsinki.

## Consent

Before enrollment in the study, all patients were thoroughly informed about the study's protocol and provided written informed consent.

## Conflicts of Interest

The authors declare no conflicts of interest.

## Supporting information


**Supplementary Figure 1:** Association between the 6‐minute walking test and Endothelium Quality Index. 6mwt: 6‐minute walking test, EQI: Endothelium Quality Index.

Supporting information.

Supporting information.

## Data Availability

Data can be accessed by contacting the corresponding author.

## References

[1] K. Neubauer and B. Zieger , “Endothelial Cells and Coagulation,” Cell and Tissue Research 387, no. 3 (2022): 391–398, 10.1007/s00441-021-03471-2. 34014399 PMC8975780

[2] N. Herrera‐Zelada , U. Zuñiga‐Cuevas , A. Ramirez‐Reyes , S. Lavandero , and J. A. Riquelme , “Targeting the Endothelium to Achieve Cardioprotection,” Frontiers in Pharmacology 12 (2021): 636134, 10.3389/fphar.2021.636134. 33603675 PMC7884828

[3] G. Heusch , “Myocardial Ischaemia‐Reperfusion Injury and Cardioprotection in Perspective,” Nature Reviews Cardiology 17, no. 12 (2020): 773–789, 10.1038/s41569-020-0403-y. 32620851

[4] T. Rassaf , M. Totzeck , U. B. Hendgen‐Cotta , S. Shiva , G. Heusch , and M. Kelm , “Circulating Nitrite Contributes to Cardioprotection by Remote Ischemic Preconditioning,” Circulation Research 114, no. 10 (2014): 1601–1610, 10.1161/CIRCRESAHA.114.303822. 24643960

[5] I. Andreadou , E. K. Iliodromitis , D. Farmakis , and D. T. Kremastinos , “To Prevent, Protect and Save the Ischemic Heart: Antioxidants Revisited,” Expert Opinion on Therapeutic Targets 13, no. 8 (2009): 945–956, 10.1517/14728220903039698. 19534573

[6] B. Ooi , B. Goh , and W. Yap , “Oxidative Stress in Cardiovascular Diseases: Involvement of Nrf2 Antioxidant Redox Signaling in Macrophage Foam Cells Formation,” International Journal of Molecular Sciences 18, no. 11 (2017): 2336, 10.3390/ijms18112336. 29113088 PMC5713305

[7] D. Lee , R. R. Pate , C. J. Lavie , X. Sui , T. S. Church , and S. N. Blair , “Leisure‐Time Running Reduces All‐Cause and Cardiovascular Mortality Risk,” Journal of the American College of Cardiology 64, no. 5 (2014): 472–481, 10.1016/j.jacc.2014.04.058. 25082581 PMC4131752

[8] C. J. Lavie , C. Ozemek , S. Carbone , P. T. Katzmarzyk , and S. N. Blair , “Sedentary Behavior, Exercise, and Cardiovascular Health,” Circulation Research 124, no. 5 (2019): 799–815, 10.1161/CIRCRESAHA.118.312669. 30817262

[9] A. Rozanski , J. A. Blumenthal , A. L. Hinderliter , S. Cole , and C. J. Lavie , “Cardiology and Lifestyle Medicine,” Progress in Cardiovascular Diseases 77 (2023): 4–13, 10.1016/j.pcad.2023.04.004. 37059409

[10] D. M. Lloyd‐Jones , Y. Hong , D. Labarthe , et al., “Defining and Setting National Goals for Cardiovascular Health Promotion and Disease Reduction: The American Heart Association's Strategic Impact Goal through 2020 and Beyond,” Circulation 121, no. 4 (2010): 586–613, 10.1161/CIRCULATIONAHA.109.192703. 20089546

[11] M. Yan , B. Hu , L. A. Tse , et al., “Behavioral Counseling for Cardiovascular Disease Prevention in 36 Low‐Income and Middle‐Income Countries,” Preventive Medicine 185 (2024): 108009, 10.1016/j.ypmed.2024.108009. 38797263

[12] D. M. Lloyd‐Jones , N. B. Allen , C. A. M. Anderson , et al., “Life's Essential 8: Updating and Enhancing the American Heart Association's Construct of Cardiovascular Health: A Presidential Advisory From the American Heart Association,” Circulation 146, no. 5 (2022): e18–e43, 10.1161/CIR.0000000000001078. 35766027 PMC10503546

[13] K. Lechner , C. von Schacky , A. L. McKenzie , et al., “Lifestyle Factors and High‐Risk Atherosclerosis: Pathways and Mechanisms Beyond Traditional Risk Factors,” European Journal of Preventive Cardiology 27, no. 4 (2020): 394–406, 10.1177/2047487319869400. 31408370 PMC7065445

[14] N. Baranwal , P. K. Yu , and N. S. Siegel , “Sleep Physiology, Pathophysiology, and Sleep Hygiene,” Progress in Cardiovascular Diseases 77 (2023): 59–69, 10.1016/j.pcad.2023.02.005. 36841492

[15] R. Allada and J. Bass , “Circadian Mechanisms in Medicine,” New England Journal of Medicine 384, no. 6 (2021): 550–561, 10.1056/NEJMra1802337. 33567194 PMC8108270

[16] J. Lin , H. Kuang , J. Jiang , et al., “Circadian Rhythms in Cardiovascular Function: Implications for Cardiac Diseases and Therapeutic Opportunities,” Medical Science Monitor: International Medical Journal of Experimental and Clinical Research 29 (2023): e942215, 10.12659/MSM.942215. 37986555 PMC10675984

[17] M. G. Figueiro and D. Pedler , “Cardiovascular Disease and Lifestyle Choices: Spotlight on Circadian Rhythms and Sleep,” Progress in Cardiovascular Diseases 77 (2023): 70–77, 10.1016/j.pcad.2023.02.004. 36841493 PMC10225333

[18] M. Yaprak , A. Altun , A. Vardar , M. Aktoz , S. Ciftci , and G. Ozbay , “Decreased Nocturnal Synthesis of Melatonin in Patients With Coronary Artery Disease,” International Journal of Cardiology 89, no. 1 (2003): 103–107, 10.1016/S0167-5273(02)00461-8. 12727015

[19] Y. Jia , D. Guo , L. Sun , et al., “Self‐Reported Daytime Napping, Daytime Sleepiness, and Other Sleep Phenotypes in the Development of Cardiometabolic Diseases: A Mendelian Randomization Study,” European Journal of Preventive Cardiology 29, no. 15 (2022): 1982–1991, 10.1093/eurjpc/zwac123. 35707994

[20] R. A. Byrne , X. Rossello , J. J. Coughlan , et al., “2023 ESC Guidelines for the Management of Acute Coronary Syndromes,” European Heart Journal 44, no. 38 (2023): 3720–3826, 10.1093/eurheartj/ehad191. 37622654

[21] S. Charfeddine , H. Ibn Hadj Amor , J. Jdidi , et al., “Long COVID 19 Syndrome: Is It Related to Microcirculation and Endothelial Dysfunction? Insights From TUN‐EndCOV Study,” Frontiers in Cardiovascular Medicine 8 (2021): 745758, 10.3389/fcvm.2021.745758. 34917659 PMC8670225

[22] M. Naghavi , A. A. Yen , A. W. Lin , H. Tanaka , and S. Kleis , “New Indices of Endothelial Function Measured by Digital Thermal Monitoring of Vascular Reactivity: Data From 6084 Patients Registry,” International Journal of Vascular Medicine 2016 (2016): 1348028, 10.1155/2016/1348028. 27830091 PMC5088311

[23] J. B. Webster , D. F. Kripke , S. Messin , D. J. Mullaney , and G. Wyborney , “An Activity‐Based Sleep Monitor System for Ambulatory Use,” Sleep 5, no. 4 (1982): 389–399, 10.1093/sleep/5.4.389. 7163726

[24] C. Smyth , “The Pittsburgh Sleep Quality Index (PSQI),” Journal of Gerontological Nursing 25, no. 12 (1999): 10, 10.3928/0098-9134-19991201-10. 10711108

[25] B. Pavy , A. Tisseau , and M. Caillon , “Le patient coronarien six mois après la réadaptation cardiaque: recherche sur l’évaluation de la réadaptation (étude RER),” Annales de Cardiologie et d'Angéiologie 60, no. 5 (2011): 252–258, 10.1016/j.ancard.2011.08.004. 21907321

[26] J. J. Kandhai‐Ragunath , C. J. M. Doggen , H. T. Jørstad , et al., “Disfunción endotelial tras infarto de miocardio con elevación del segmento ST y evolución a largo plazo: un estudio con tonometría arterial periférica e hiperemia reactiva,” Revista española de cardiología 69, no. 7 (2016): 664–671, 10.1016/j.rec.2015.12.020. 27068872

[27] J. D. Allbritton‐King and G. García‐Cardeña , “Endothelial Cell Dysfunction in Cardiac Disease: Driver or Consequence?,” Frontiers in Cell and Developmental Biology 11 (2023): 1278166, 10.3389/fcell.2023.1278166. 37965580 PMC10642230

[28] P. Poredos , A. V. Poredos , and I. Gregoric , “Endothelial Dysfunction and Its Clinical Implications,” Angiology 72, no. 7 (2021): 604–615, 10.1177/0003319720987752. 33504167

[29] T. Z. Liu , C. Xu , M. Rota , et al., “Sleep Duration and Risk of All‐Cause Mortality: A Flexible, Non‐Linear, Meta‐Regression of 40 Prospective Cohort Studies,” Sleep Medicine Reviews 32 (2017): 28–36, 10.1016/j.smrv.2016.02.005. 27067616

[30] V. N. Jaspan , G. S. Greenberg , S. Parihar , et al., “The Role of Sleep in Cardiovascular Disease,” Current Atherosclerosis Reports 26, no. 7 (2024): 249–262, 10.1007/s11883-024-01207-5. 38795275 PMC11192677

[31] M. Behl , E. Veledar , A. Quyyumi , et al., “Vascular Endothelial Function and Self‐Reported Sleep,” The American Journal of the Medical Sciences 347, no. 6 (2014): 425–428, 10.1097/MAJ.0b013e31829bc950. 23842206 PMC3852200

[32] B. J. Holmer , S. S. Lapierre , D. E. Jake‐Schoffman , and D. D. Christou , “Effects of Sleep Deprivation on Endothelial Function in Adult Humans: A Systematic Review,” GeroScience 43, no. 1 (2021): 137–158, 10.1007/s11357-020-00312-y. 33558966 PMC8050211

[33] C. Dibner , U. Schibler , and U. Albrecht , “The Mammalian Circadian Timing System: Organization and Coordination of Central and Peripheral Clocks,” Annual Review of Physiology 72 (2010): 517–549, 10.1146/annurev-physiol-021909-135821. 20148687

[34] B. D. Goldman , “Mammalian Photoperiodic System: Formal Properties and Neuroendocrine Mechanisms of Photoperiodic Time Measurement,” Journal of Biological Rhythms 16, no. 4 (2001): 283–301, 10.1177/074873001129001980. 11506375

[35] R. J. Reiter , Q. Ma , and R. Sharma , “Melatonin in Mitochondria: Mitigating Clear and Present Dangers,” Physiology (Bethesda, Md.) 35, no. 2 (2020): 86–95, 10.1152/physiol.00034.2019. 32024428

[36] A. Domínguez‐Rodríguez , P. Abreu‐González , N. Báez‐Ferrer , R. J. Reiter , P. Avanzas , and D. Hernández‐Vaquero , “Melatonin and Cardioprotection in Humans: A Systematic Review and Meta‐Analysis of Randomized Controlled Trials,” Frontiers in Cardiovascular Medicine 8 (2021): 635083, 10.3389/fcvm.2021.635083. 34055929 PMC8149621

[37] D. Mozaffarian , T. Hao , E. B. Rimm , W. C. Willett , and F. B. Hu , “Changes in Diet and Lifestyle and Long‐Term Weight Gain in Women and Men,” New England Journal of Medicine 364, no. 25 (2011): 2392–2404, 10.1056/NEJMoa1014296. 21696306 PMC3151731

[38] E. J. Vandercappellen , A. Koster , H. H. C. M. Savelberg , et al., “Sedentary Behaviour and Physical Activity Are Associated With Biomarkers of Endothelial Dysfunction and Low‐Grade Inflammation—Relevance for (Pre)Diabetes: The Maastricht Study,” Diabetologia 65, no. 5 (2022): 777–789, 10.1007/s00125-022-05651-3. 35119485 PMC8960649

[39] I. Bellos , S. Marinaki , P. Lagiou , et al., “Association of Physical Activity With Endothelial Dysfunction Among Adults With and Without Chronic Kidney Disease: The Maastricht Study,” Atherosclerosis 383 (2023): 117330, 10.1016/j.atherosclerosis.2023.117330. 37837705

[40] S. S. Thosar , B. D. Johnson , J. D. Johnston , and J. P. Wallace , “Sitting and Endothelial Dysfunction: The Role of Shear Stress,” Medical Science Monitor: International Medical Journal of Experimental and Clinical Research 18, no. 12 (2012): 173–180, 10.12659/msm.883589. PMC3560806 23197245

[41] B. M. F. M. Duvivier , J. E. Bolijn , A. Koster , C. G. Schalkwijk , H. H. C. M. Savelberg , and N. C. Schaper , “Reducing Sitting Time Versus Adding Exercise: Differential Effects on Biomarkers of Endothelial Dysfunction and Metabolic Risk,” Scientific Reports 8, no. 1 (2018): 8657, 10.1038/s41598-018-26616-w. 29872225 PMC5988819

[42] C. J. Lavie , R. Arena , D. L. Swift , et al., “Exercise and the Cardiovascular System: Clinical Science and Cardiovascular Outcomes,” Circulation Research 117, no. 2 (2015): 207–219, 10.1161/CIRCRESAHA.117.305205. 26139859 PMC4493772

[43] K. S. Early , A. Stewart , N. Johannsen , C. J. Lavie , J. R. Thomas , and M. Welsch , “The Effects of Exercise Training on Brachial Artery Flow‐Mediated Dilation: A Meta‐Analysis,” Journal of Cardiopulmonary Rehabilitation and Prevention 37, no. 2 (2017): 77–89, 10.1097/HCR.0000000000000206. 28033167

[44] G. Grandes , A. García‐Alvarez , M. Ansorena , et al., “Any Increment in Physical Activity Reduces Mortality Risk of Physically Inactive Patients: Prospective Cohort Study in Primary Care,” British Journal of General Practice 73, no. 726 (2023): e52–e58, 10.3399/BJGP.2022.0118. PMC9639597 36316160

[45] Y. Y. Liang , H. Feng , Y. Chen , et al., “Joint Association of Physical Activity and Sleep Duration With Risk of All‐Cause and Cause‐Specific Mortality: A Population‐Based Cohort Study Using Accelerometry,” European Journal of Preventive Cardiology 30, no. 9 (2023): 832–843, 10.1093/eurjpc/zwad060. 36990109

[46] M. Banach , J. Lewek , S. Surma , et al., “The Association Between Daily Step Count and All‐Cause and Cardiovascular Mortality: A Meta‐Analysis,” European Journal of Preventive Cardiology 30, no. 18 (2023): 1975–1985, 10.1093/eurjpc/zwad229. 37555441

[47] A. Rozanski , “New Principles, the Benefits, and Practices for Fostering a Physically Active Lifestyle,” Progress in Cardiovascular Diseases 77 (2023): 37–49, 10.1016/j.pcad.2023.04.002. 37030619

[48] T. Yamaji , T. Harada , Y. Hashimoto , et al., “Inconvenient Relationship of Haemoglobin A1c Level With Endothelial Function in Type 2 Diabetes in a Cross‐Sectional Study,” BMJ Open 11, no. 6 (2021): e045415, 10.1136/bmjopen-2020-045415. PMC8191618 34108164

[49] E. Dąbrowska and K. Narkiewicz , “Hypertension and Dyslipidemia: The Two Partners in Endothelium‐Related Crime,” Current Atherosclerosis Reports 25, no. 9 (2023): 605–612, 10.1007/s11883-023-01132-z. 37594602 PMC10471742

[50] Y. Higashi , “Smoking Cessation and Vascular Endothelial Function,” Hypertension Research 46, no. 12 (2023): 2670–2678, 10.1038/s41440-023-01455-z. 37828134 PMC10695829

[51] A. J. Donato , D. R. Machin , and L. A. Lesniewski , “Mechanisms of Dysfunction in the Aging Vasculature and Role in Age‐Related Disease,” Circulation Research 123, no. 7 (2018): 825–848, 10.1161/CIRCRESAHA.118.312563. 30355078 PMC6207260

[52] U. Landmesser , S. Dikalov , S. R. Price , et al., “Oxidation of Tetrahydrobiopterin Leads to Uncoupling of Endothelial Cell Nitric Oxide Synthase in Hypertension,” Journal of Clinical Investigation 111, no. 8 (2003): 1201–1209, 10.1172/JCI14172. 12697739 PMC152929

[53] G. Patti , V. Pasceri , R. Melfi , et al., “Impaired Flow‐Mediated Dilation and Risk of Restenosis in Patients Undergoing Coronary Stent Implantation,” Circulation 111, no. 1 (2005): 70–75, 10.1161/01.CIR.0000151308.06673.D2. 15630038

[54] M. Guazzi , G. Reina , P. Gripari , G. Tumminello , M. Vicenzi , and R. Arena , “Prognostic Value of Flow‐Mediated Dilatation Following Myocardial Infarction,” International Journal of Cardiology 132, no. 1 (2009): 45–50, 10.1016/j.ijcard.2007.10.036. 18180054

[55] R. Ross , R. Arena , J. Myers , P. Kokkinos , and L. A. Kaminsky , “Update to the 2016 American Heart Association Cardiorespiratory Fitness Statement,” Progress in Cardiovascular Diseases 83 (2024): 10–15, 10.1016/j.pcad.2024.02.003. 38387825

[56] R. Ross , S. N. Blair , R. Arena , et al., “Importance of Assessing Cardiorespiratory Fitness in Clinical Practice: A Case for Fitness as a Clinical Vital Sign: A Scientific Statement From the American Heart Association,” Circulation 134, no. 24 (2016): e653–e699, 10.1161/CIR.0000000000000461. 27881567

[57] E. Z. Kabbach , A. D. Heubel , C. da Luz Goulart , et al., “Association of Exercise Capacity and Endothelial Function in Patients With Severe Exacerbations of Chronic Obstructive Pulmonary Disease,” Scientific Reports 11, no. 1 (2021): 461, 10.1038/s41598-020-80601-w. 33432116 PMC7801495

[58] F. Suhr , S. Gehlert , M. Grau , and W. Bloch , “Skeletal Muscle Function during Exercise—Fine‐Tuning of Diverse Subsystems by Nitric Oxide,” International Journal of Molecular Sciences 14, no. 4 (2013): 7109–7139, 10.3390/ijms14047109. 23538841 PMC3645679

[59] Y. H. Hong , A. C. Betik , and G. K. McConell , “Role of Nitric Oxide in Skeletal Muscle Glucose Uptake During Exercise,” Experimental Physiology 99, no. 12 (2014): 1569–1573, 10.1113/expphysiol.2014.079202. 25192731

[60] C. Özcan , A. Deleskog , A. M. Schjerning Olsen , H. Nordahl Christensen , M. Lock Hansen , and G. Hilmar Gislason , “Coronary Artery Disease Severity and Long‐Term Cardiovascular Risk in Patients With Myocardial Infarction: A Danish Nationwide Register‐Based Cohort Study,” European Heart Journal ‐ Cardiovascular Pharmacotherapy 4, no. 1 (2018): 25–35, 10.1093/ehjcvp/pvx009. 28444162 PMC5843132

[61] J. Hung , T. K. Teng , J. Finn , et al., “Trends From 1996 to 2007 in Incidence and Mortality Outcomes of Heart Failure After Acute Myocardial Infarction: A Population‐Based Study of 20 812 Patients With First Acute Myocardial Infarction in Western Australia,” Journal of the American Heart Association 2 (2013): e000172, 10.1161/JAHA.113.000172. 24103569 PMC3835218

[62] S. D. Katz , K. Hryniewicz , I. Hriljac , et al., “Vascular Endothelial Dysfunction and Mortality Risk in Patients With Chronic Heart Failure,” Circulation 111, no. 3 (2005): 310–314, 10.1161/01.CIR.0000153349.77489.CF. 15655134

[63] Y. H. Kim , T. Kitai , R. Morales , K. Kiefer , T. Chaikijurajai , and W. H. W. Tang , “Usefulness of Serum Biomarkers of Endothelial Glycocalyx Damage in Prognosis of Decompensated Patients With Heart Failure with Reduced Ejection Fraction,” The American Journal of Cardiology 176 (2022): 73–78, 10.1016/j.amjcard.2022.04.036. 35606171

[64] S. Dimitropoulos , V. C. Mystakidi , E. Oikonomou , et al., “Association of Soluble Suppression of Tumorigenesis‐2 (ST2) With Endothelial Function in Patients With Ischemic Heart Failure,” International Journal of Molecular Sciences 21, no. 24 (2020): 9385, 10.3390/ijms21249385. 33317161 PMC7764062

