# Supplementary figures and images for: Endothelial Dysfunction in Acute Myocardial Infarction: A Complex Association With Sleep Health, Traditional Cardiovascular Risk Factors and Prognostic Markers
Source: Clin Cardiol. 2025 Jan 28;48(1):e70080. doi: 10.1002/clc.70080 (PMC11773158; doi:10.1002/clc.70080)

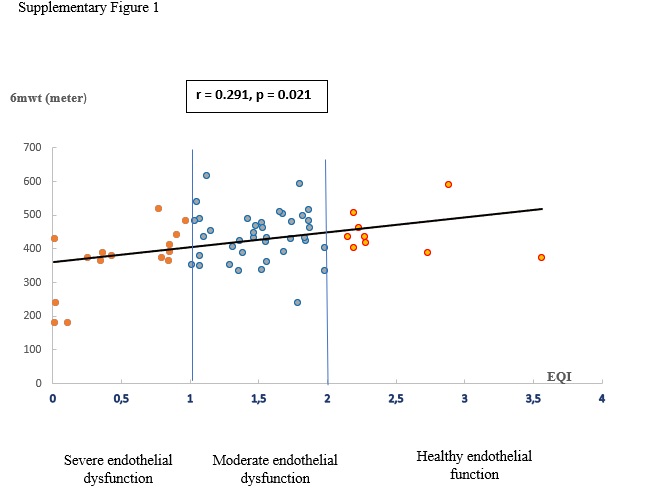

Supplement: Supplementary file 1 — Supplementary Figure 1: Association between the 6‐minute walking test and Endothelium Quality Index. 6mwt: 6‐minute walking test, EQI: Endothelium Quality Index. [file CLC-48-e70080-s003.jpg]
