# Supplementary material for: Endothelial Dysfunction in Acute Myocardial Infarction: A Complex Association With Sleep Health, Traditional Cardiovascular Risk Factors and Prognostic Markers
Source: Clin Cardiol. 2025 Jan 28;48(1):e70080. doi: 10.1002/clc.70080 (PMC11773158; doi:10.1002/clc.70080)
Supplement: Supplementary file 2 — Supporting information. [file CLC-48-e70080-s002.docx]

**Supplementary Table 1: Multiple regression analysis of cardiovascular risk factors and endothelial function.**

| **Regression Analysis: Adjusted R^2^ = 0.500, F = 8.749, p <0.001** | | | | | |
| --- | --- | --- | --- | --- | --- |
| **Variable** | **B** | **SE** | **β** | **T** | ***P*** |
| Smoking | −0.007 | 0.002 | −0.288 | −2.922 | 0.005 |
| Body mass index | −0.035 | 0.19 | −0.180 | −1.828 | 0.073 |
| Age | −0.037 | 0.009 | −0.428 | −4.281 | <0.001 |
| Triglyceride | -0.107 | 0.113 | -0.093 | -1.342 | 0.349 |
| Glycated hemoglobin | -0.050 | 0.037 | -0.134 | -1.342 | 0.185 |
| Hypertension | -0.11 | 0.140 | -0.007 | -0.750 | 0.941 |
| Family history of coronary artery diseases | -0.031 | 0.137 | -0.022 | -0.228 | 0.821 |
| Physical activity levels | 0.117 | 0.023 | 0.476 | 5.186 | <0.001 |

| **Regression Analysis: Adjusted R^2^ = 0.504, F = 16.741, *p* <0.001** | | | | | |
| --- | --- | --- | --- | --- | --- |
| **Variable** | **B** | **SE** | **β** | **T** | ***P*** |
| Smoking | −0.007 | 0.002 | −0.313 | −3.373 | 0.001 |
| Age | −0.042 | 0.008 | −0.476 | −5.135 | <0.001 |
| Physical activity levels | 0.128 | 0.022 | 0.520 | 5.786 | <0.001 |

B = unstandardized regression coefficient, β = standardized coefficient, p = level of statistical significance, R = multiple correlation coefficient, R2 = proportion of variance, SE = standard error, t = t statistic.
