# Supplementary material for: Endothelial Dysfunction in Acute Myocardial Infarction: A Complex Association With Sleep Health, Traditional Cardiovascular Risk Factors and Prognostic Markers
Source: Clin Cardiol. 2025 Jan 28;48(1):e70080. doi: 10.1002/clc.70080 (PMC11773158; doi:10.1002/clc.70080)
Supplement: Supplementary file 3 — Supporting information. [file CLC-48-e70080-s001.docx]

**Supplementary Table 2: Multiple regression: effects of endothelial dysfunction on cardiorespiratory fitness, coronary artery disease severity, and left ventricle ejection fraction.**

| **Regression Analysis: R2 = 0.096, F = 3.185, p=0.03** | | | | | |
| --- | --- | --- | --- | --- | --- |
| **Variable** | **B** | **SE** | **β** | **T** | ***p*** |
| Left ventricle ejection fraction | 0.007 | 0.1 | 0.89 | 0.716 | 0.477 |
| Coronary artery disease severity | 0.013 | 0.126 | 0.13 | 0.101 | 0.920 |
| Cardiorespiratory fitness | 0.003 | 0.001 | 0.363 | 3.005 | 0.004 |

B = unstandardized regression coefficient; β = standardized coefficient; p = level of statistical significance; R = multiple correlation coefficient; R2 = proportion of variance; SE = standard error; t = t statistic.
